# Horse Housing on Prince Edward Island, Canada: Attitudes and Experiences Related to Keeping Horses Outdoors and in Groups

**DOI:** 10.3390/ani13020275

**Published:** 2023-01-13

**Authors:** Megan Ross, Kathryn Proudfoot, Katrina Merkies, Ibrahim Elsohaby, Molly Mills, Kathleen Macmillan, Shawn Mckenna, Caroline Ritter

**Affiliations:** 1 Department of Health Management, Atlantic Veterinary College, University of Prince Edward Island, Charlottetown, PE C1A 4P3, Canada; 2 Department of Animal Biosciences, University of Guelph, Guelph, ON N1G 2W1, Canada; 3 Campbell Centre for the Study of Animal Welfare, Guelph, ON N1G 2W1, Canada; 4 Department of Infectious Diseases and Public Health, Jockey Club College of Veterinary Medicine and Life Sciences, City University of Hong Kong, Hong Kong SAR 999077, China

**Keywords:** horse management, horse owner perceptions, motivators and barriers, horse care

## Abstract

**Simple Summary:**

The decisions of horse owners can profoundly affect horse welfare, but little research has incorporated horse owner voices within the academic literature. This project aimed to understand horse owner attitudes and experiences related to horse welfare in different housing contexts. In regard to horse housing, owners considered their horses’ mental state, nutrition, behavior, environment, and health. Further, they believed that horse welfare is better in outdoor and group housing but showed less agreement that the standard of care for horses in those housing types is better than indoor and individual housing. Horse owners who kept their horses indoors part-time and individually were more likely to agree that the standard of care for horses is better in these housing types. Overall, owner attitudes corresponded with the way they housed their horses, and they considered different horse welfare aspects in their housing decisions.

**Abstract:**

Limited research has assessed the “human dimension” of horse care. The aims of this study were to (1) understand horse owner attitudes toward horse welfare when kept outdoors versus indoors and in groups versus individually, (2) compare horse owner attitudes toward horse welfare with the ways in which they house their horses, and (3) explore horse owner reasons for and challenges with their horses’ housing. Seventy-six horse owners in Prince Edward Island, Canada completed a questionnaire. Non-parametric tests and quantitative content analysis were used for data analysis. Consistent with the way horses were kept, most (82–96%) owners agreed that horses’ physical health, mental well-being, and natural living were better when kept outdoors and in groups. Fewer (64–68%) participants agreed that the horses’ standard of care was better when kept outdoors or in groups. Results show associations between owners whose attitudes suggest indoor and/or individual housing is better for horse welfare and keeping their horses indoors part-time and/or individually. Two overarching themes were developed from owners’ responses regarding their reasons and challenges related to the ways in which horses were housed: horse-centered and owner-centered care. The results indicate that horse owners’ choices about their horses’ housing correspond to beliefs about improved horse welfare.

## 1. Introduction

Horse owner care practices contribute to horse welfare challenges worldwide [1,2,3]. Understanding owners’ attitudes toward the ways in which horses should be kept may provide insights into barriers that prevent evidence-based management practices. For example, for human convenience or due to fear of injury [4,5], the accepted standards of care afforded to horses include confinement to a stall [6], the inability to graze [1], and social isolation [7,8,9,10]. These practices are far removed from the way horses evolved to spend many hours moving and grazing each day [9,11,12]. Therefore, it has been reported and encouraged in the literature that owners should keep their horses in more natural environments to improve their welfare, such as in outdoor groups rather than indoor individual stalls [8,10,13]. 

There are three interrelated components of animal welfare highlighted by Fraser et al. (1997) [14] that include the animal’s ability to live a natural life, the animal’s basic health and functioning (i.e., physical health), and the animal’s affective states (i.e., mental well-being). Further, Weary and Robbins (2019) [15] reflected on the public’s expectation for humane care, and the often-related assumption that humans have a duty of care toward domesticated animals. Hence, the concept of “standard of care” has been discussed as a component in animal welfare publications [16,17,18,19], and includes the quality, appropriateness, or humaneness of care provided to the animals [19]. Although undoubtedly related to the three welfare components highlighted by Fraser et al. (1997) [14], the concept of standard of care specifically highlights the human–animal interaction dimension of animal welfare [15]. Using concepts related to horses’ physical health, mental well-being, natural living, and standard of care in welfare assessments will provide a basis to support horse owner decision-making. Social science research within the equestrian community is an emerging field, and understanding horse owner attitudes, perceptions, and experiences in daily practice is an essential component when considering the care for horses [20,21,22,23].

Little is known about the housing of horses in Canada as well as owners’ attitudes toward specific living conditions [24,25,26,27]. Therefore, this project aims to contribute a deeper understanding of horse owners’ attitudes toward housing-related decisions. This project has an overarching aim to incorporate recreational horse owner voices within research. Specific objectives for this project were to (1) understand horse owner attitudes toward horse welfare when kept indoors versus outdoors and individually versus in groups; (2) assess whether horse owner attitudes toward horse welfare in different living conditions align with the way they keep their horses, (3) explore horse owner attitudes toward and experiences with their horses’ living conditions.

## 2. Materials and Methods

### 2.1. Questionnaire Development

The questionnaire (Supplement Questionnaire S1) was created using Qualtrics software, Version 6/22 (Qualtrics, Provo, UT, USA). An interdisciplinary team with experience in animal welfare, horse management, veterinary medicine, and social sciences developed the questionnaire. The questionnaire was pilot tested with six horse owners whose answers were not included in the analysis. Between 10 to 30 min were required by the participants to complete the questionnaire. 

The questionnaire consisted of the following six sections: 

Section 1: Horse owner demographics. This section included two questions related to horse owners’ gender and birth year, and one multiple choice question assessing owner education level.

Section 2: Horse owner welfare attitudes toward different housing types. Horse owners’ level of agreement with 8 statements related to horse welfare was measured using 7-point Likert items (strongly disagree (1) to strongly agree (7)). Four welfare concerns were addressed: the horse’s physical health, mental well-being, ability to live a natural life, and standard of care. There was one statement per welfare concern for two specific living conditions: indoor versus outdoor living and group versus individual living. Thus, for indoor versus outdoor living conditions, the statements read: “The horse’s (1. physical health, 2. mental well-being, 3. ability to live a natural life, 4. standard of care) is better when housed outdoors versus indoors”. For individual versus group living conditions, the statements read: “The horse’s (1. physical health, 2. mental well-being, 3. ability to live a natural life, 4. standard of care) is better when horses are housed individually versus in groups”.

Section 3: Horse care and management. The third section gathered information about the number of horses that participants owned, whether owners cared for or managed horses they did not own, the level of decision-making capacity they had regarding their horses’ housing, and the information sources they used to assist in their decisions related to their horses’ housing. 

Section 4: Horse demographics. The fourth section included open-ended and multiple-choice questions about the horses’ age, discipline, and feeding. 

Section 5: Horse housing type. Horse owners were asked to describe the ways in which their horses were predominantly housed in the last four weeks using multiple choice questions. Since some horses with the same owner may be kept differently, owners were only asked to describe housing for up to five horses on each farm. Housing types were classified as outdoor pasture/paddock, indoor housing attached to pasture/paddock/run, indoor housing, moved between indoor housing and pasture/paddock, and moved between indoor housing attached to outdoor run and pasture/paddock. Specific follow-up questions were asked based on the type of housing horse owners indicated. 

Section 6: Owner experience when caring for their horse. In this section, the first open-ended question asked owners to describe specific reasons for housing horses the way they do. Second, owners were asked to describe specific challenges they face when housing their horse (open-ended question). Third, owners were asked about their level of satisfaction with their horses’ current housing type, which was measured using a 5-point semantic differential item (strongly dissatisfied (1) to strongly satisfied (5)). If horse owners were less than strongly satisfied, they were presented with the third open-ended question asking about changes they would like to make to their horse’s current housing. 

### 2.2. Participant Recruitment

Data were collected between 25 August 2021 and 29 November 2021. The study was advertised through posters in local feed stores, tack shops, and horse veterinary clinics in Prince Edward Island (PEI) and one live interview through CBC radio. Sixty horse farms voluntarily participated, and 76 horse owners filled out the questionnaire on-farm using an electronic tablet. 

### 2.3. Data Analysis

#### 2.3.1. Horse Owner Attitudes: Indoor Versus Outdoor and Individual versus Group Living

Stata/BE 17.0 was used for statistical analysis. For analysis, we dichotomized the five housing types into outdoor housing full-time and indoor housing part-time (i.e., horses were housed indoors for at least part of a 24 h period). One horse in this study was housed indoors full-time based on owner preference (not related to medical reasons). Further, horse housing was dichotomized into individual and group housing. Group housing included horses housed with other hoofed animals and horses housed with both other hoofed animals and horses.

The Friedman, non-parametric two-way ANOVA was used to compare differences in horse owners’ level of agreement between the four statements for indoor versus outdoor and four statements for individual versus group housing. A Bonferroni adjustment for multiple comparisons was applied; hence, for these comparisons, a *p*-value of <0.0083 (0.05 divided by 6 statistical comparisons) was considered statistically significant. 

The Wilcoxon rank-sum test was used to compare horse owner attitudes toward horse welfare in different housing types (i.e., outdoor versus indoors and individual versus group) with whether their horses were housed outdoors full-time or indoors part-time and in groups versus individually. Horse welfare was measured using the three concepts of animal welfare [14] and standard of care. Bonferroni adjustment for multiple comparisons was applied; hence, a *p*-value of <0.0125 (0.05 divided by 4 statistical comparisons) was considered statistically significant for these comparisons. 

#### 2.3.2. Quantitative Content Analysis

Common themes from the three open-ended questions were coded inductively using quantitative content analysis [28]. Responses were coded as manifest content (i.e., responses were coded with minimal underlying interpretation from coders) to enhance replicability and lessen variation related to the underlying meaning behind responses [29]. Initial coding and theme development were discussed between the authors M.R., M.M. and C.R. Then, inter-coder reliability for developed (sub-)themes was assessed between the first author and a research assistant not involved in previous discussions using Cohen’s kappa statistic. Finally, the frequencies of developed themes were calculated. 

#### 2.3.3. Comparing Horse Owner Open-Text Responses with the Ways in Which Horses Were Kept

Using Fisher’s exact tests, the frequencies of themes and subthemes developed through quantitative content analysis were compared with the ways in which horses were housed. Each theme and subtheme were compared with housing type, adjusting for multiple comparisons using Bonferroni adjustments (i.e., here, a *p*-value of <0.01 (0.05/5) was considered statistically significant).

## 3. Results

### 3.1. Horse Owner and Horse Demographics

Farms that participated were located throughout PEI. The horses in the study were used for various purposes such as breeding, leisure, school horses, or retirement, with an age range from less than 1 to 34 years (Table 1). Nearly all horses (n = 211) that were owned by one of the participants had outdoor access for at least part of the day. Most commonly, horses were kept in full-time outdoor group housing (79% of horses) or kept individually indoor part-time but in groups when outdoors (15% of horses). Most (68%) horse owners indicated that they did not take care of horses they did not own. Further, most (87%) participants identified as women, and there was a wide age range from 21 to 81 years (Table 1). Horse owners predominantly used their veterinarian, other horse caretakers in their community, and their farrier as their primary information sources for housing- and feeding-related decisions (Table 1). 

### 3.2. Horse Owner Attitudes toward Horse Welfare: Indoor versus Outdoor Housing 

**Figure 1:**
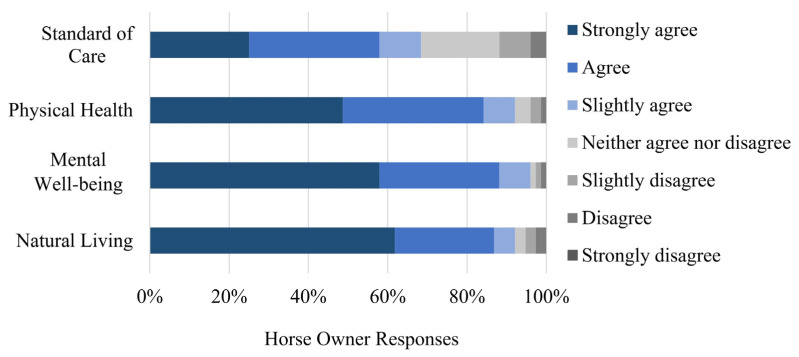
Owners’ level of agreement with horses’ welfare being better when housed outdoors versus indoors.

Almost all participants agreed to some extent (i.e., slightly agreed, agreed, strongly agreed) that horses’ physical health (93% agreement), mental well-being (96% agreement), and ability to live a natural life (92% agreement) are better when they are housed outdoors versus indoors (Figure 1). Comparatively, fewer (69%) horse owners agreed to some extent that horses’ standard of care is better when housed outdoors than when they are housed indoors. The Friedman two-way ANOVA test showed significant differences between owner responses to the statement related to the standard of care and the other three welfare statements: physical health (*p* < 0.001), mental well-being (*p* < 0.001), and ability to live naturally (*p* < 0.001). The Friedman two-way ANOVA test did not show significant differences between responses to the three welfare statements related to physical health, mental well-being, and ability to live naturally (*p* > 0.008 for each comparison).

After the Bonferroni adjustment, horse owners’ level of agreement with the statements related to mental well-being (*p* = 0.10), physical health (*p* = 0.02), and ability to live a natural life (*p* = 0.03) were not associated with whether they housed their horses outdoors full-time or indoors part-time. However, horse owners who housed their horses indoors part-time indicated higher levels of agreement to the statement that the standard of care provided to horses was better in indoor versus outdoor housing (*p* = 0.007). 

### 3.3. Horse Owner Attitudes toward Horse Welfare: Individual versus Group Housing

**Figure 2:**
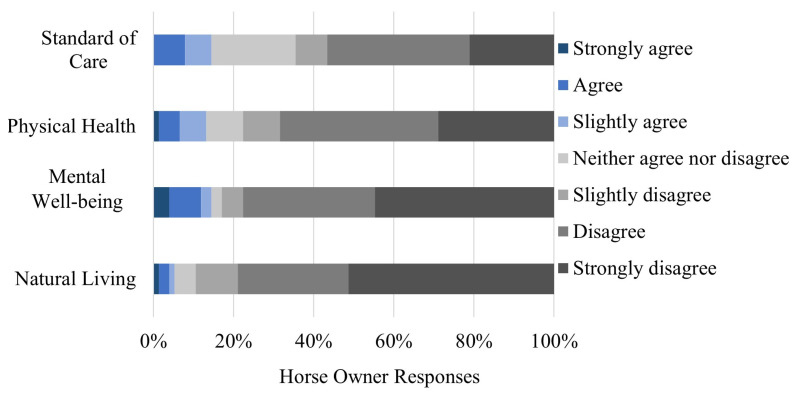
Owners’ level of agreement with horses’ welfare being better when housed individually versus in groups.

Most participants disagreed to some extent (i.e., slightly disagreed, disagreed, strongly disagreed) that horses’ physical health (77% disagreement), mental well-being (83% disagreement), and ability to live a natural life (88% disagreement) are better when they are housed individually versus in groups (Figure 2). Comparatively, a lower percentage (65%) of horse owners disagreed to some extent that a horse’s standard of care is better when housed individually than when they are housed in groups. The Friedman two-way ANOVA test showed significant differences between owner responses to the statement related to the standard of care and the other three welfare statements: physical health (*p* = 0.004), mental well-being (*p* < 0.001), and natural living (*p* < 0.001). There were no significant differences between responses to the three other welfare statements (*p* > 0.008 for each comparison). 

Horse owners who housed their horses individually versus in groups had higher levels of agreement with the welfare statement that the horse’s “ability to live a natural life is better when housed individually rather than in groups” (*p* = 0.006), that the horse’s “physical health is better when housed individually versus in groups” (*p* = 0.007), and that horses’ standard of care is better when they are housed individually versus in groups (*p* = 0.006). There was no significant association between the owner’s level agreement with the statement that horses’ mental well-being is better when housed individually versus in groups (*p* = 0.019).

### 3.4. Horse Owner Reasons for Housing Horses in Current Conditions

Two overarching themes (i.e., owner-centered and horse-centered care) with four and five subthemes, respectively, were developed from the horse owners’ responses to the open-ended question regarding the reasons for their current horses’ housing (Table 2). 

The owner-centered care theme included reasons affecting the owner in some way or tradition (e.g., “Always the way it’s been”). Most of the responses were related to the owner’s ease of care, such as “less work”, “easy to take care of them in a pasture/paddock situation”, and “easy to feed” (Table 2). Other responses indicated the reasons for housing their horses were based on other peoples’ decisions, such as “that is how the [farm] owner has the barn layout”, “the rule is that horses must be outside”, and “availability of barn” and cost: “all I can afford”.

The horse-centered care theme included reasons that affected the horse, such as their environment (e.g., “better air quality”). The highest number of responses were coded under the subtheme of freedom to perform behavior, which included the horse’s ability to have freedom of choice, live naturally, or express natural behaviors. Owners discussed reasons for housing horses outdoors, such as it is “better for their health”, the horses had “comfort with other horses”, they had the “ability to graze naturally”, and/or live in a “natural environment”. Some owners identified specific health-related reasons for housing horses outdoors, such as “[horse has] stifle issues and can’t be in a stall”. Another horse had “lameness/arthritis issues, and she needs to keep moving”. Further, some owners indicated negative consequences related to keeping their horses in stalls. This included the belief that the horse’s legs would swell up (“stock up”) and that their horse “gets bored in a stall”.

For the horse-centered and owner-centered themes, Cohen’s kappa statistic for inter-rater reliability was 0.85 and 0.88, which are within the range of almost perfect agreement [30]. Overall, there was no association between horse owner responses (horse-centered themes or owner-centered themes) and whether horses were housed outdoors full-time or indoors part-time nor if their horses were housed individually versus in groups. 

### 3.5. Housing-Related Challenges

The second open-ended question asked horse owners to state challenges they face when housing horses in their current housing condition (Table 2). Again, responses were categorized into owner-centered and horse-centered care themes. All the owner-centered challenges were related to time commitment (“increased time to clean stalls”, “a lot of work to maintain [horses] on a daily basis”). 

Horse-centered care responses were categorized into the same themes previously created for owner reasons for keeping horses in different living conditions (i.e., freedom to perform behavior, environment, mental state, physical health, and nutrition). Two of the 23 responses within the “Freedom for Behavior” theme were related to the horse not having freedom of choice: “no free choice shelter from weather” and the horse’s ability to live naturally: “lack of natural movement”. The rest of the responses were related to the horse’s social dynamics. Specifically, some responses indicated that owners wanted horses to live in a group setting, such as the horse is “inside alone” or that their horse “is lonely as there is no other horse with her”. Other responses indicated some of the challenges related to housing horses in groups, such as “making sure horses are getting along, so nobody gets trapped in [the] shelter” or that it is “difficult to individualize grain feeding”. Environmental challenges included not having stalls in case of injury (“if someone is injured, having no stall for confinement”) or poor weather (“[horse] needs to go into a separate area in bad weather due to small space and dominant horse”), “worms and parasites”, “manure management”, “bug population” and “turnout depends on weather”. Additionally, owners mentioned nutritional and feeding challenges such as “difficult to individualize grain feeding”, separating horses when feeding (e.g., “feeding separation”), “too much grazing if left [outside],” and to “limit grass intake”.

For horse-centered care themes, Cohen’s kappa statistic for inter-rater reliability was 0.84, which is within the range of almost perfect agreement [30]. Inter-rater reliability was not used for owner-centered challenges because only one theme was developed (time commitment). There was no significant association between the themes and whether horse owners housed their horses outdoors full-time or indoors part-time, nor if their horses were housed individually versus in groups. 

### 3.6. Owners’ Desired Changes Related to Horse Housing

When owners were asked what they would change about their horses’ housing, the most common answer was upgrading the barn (n = 35; 42%). Some potential barn upgrades were related to hay storage, such as “more storage for hay”, while others included changes to the stalls and barn flooring, such as “larger stalls”, “larger barn with wider aisles”, and “higher walls”. between stalls and changing the alleyway to “cement with rubber mats”. More shelter for horses was another common theme (n = 13; 16%) which included shelter alterations such as “4-sided shelter instead of 3-sided” and adding a “new shelter”. Better maintenance of pasture and increased turnout (n = 13, 16%) were additional common changes owners wished to implement. Some owners indicated they would “clean and divide the pasture for rotation”, that they wanted “nicer grass in paddocks”, or that they wanted their horse to have “more turnout” or “more pasture”. Fencing upgrades (n = 9, 11%) included responses such as “better fences”, “upgrade fence to all-electric rope”, “more fencing in the winter”, “permanent fencing”, “safer fencing” or “more gates/easier access to fields”. Less common changes owners indicated were categorized as “Other” and included wishing their horses could have “more access to round bale to ensure no competition for food” or the horses at least “being able to have round bales”, while in other cases owners indicated they did not want round bales (e.g., “no free choice round bales ever”). Better “bug control” or being able to bring the horses “in at night”. No significant difference was detected between the themes and whether owners housed their horses outdoors full-time or indoors part-time or individually versus in groups. Despite some owners indicating specific changes they would like to implement, most horse owners (92%; n = 70) indicated that they were satisfied or strongly satisfied with their horse’s current form of housing.

## 4. Discussion

As the ultimate decision-makers for their horse’s care, horse owners are at the forefront of horse welfare. Therefore, horse owners’ experiences with and reasons for keeping their horses in different conditions should be considered paramount in the horse welfare literature. This study aims to investigate the consistency between horse owners’ attitudes and the way their horses are kept as well as to understand their experiences with housing their horses. The sample of owners in this study predominantly kept their horses outdoors full-time with the ability to socialize. Similarly, previous research reported that horses in Canada [24,25,31] as well as certain Scandinavian countries [32] are commonly kept outdoors for at least part of a 24 h period. Outdoor housing appears to be less common in other countries where issues surrounding the welfare of horses living in restricted and isolated box stalls have been reported [3,5,8,33]. 

Most horse owners agreed that their horse’s physical health, mental well-being, and natural living are better when kept outdoors and in groups. This finding is consistent with research reporting that horses kept outdoors have higher oxytocin levels [9], lower cortisol levels [13], increased musculoskeletal health [34], improved fitness [35] and display less stereotypic and abnormal repetitive behavior [5,8,36]. Further, group housing allows horses to perform natural behaviors that reduce physiological and behavioral signs of stress, such as lower counts of fecal cortisol, cooler eye temperature, and being easier to handle [37,38]. However, compared to the other welfare attributes, horse owners in this study were less likely to agree that their horse’s standard of care is better when housed outdoors or in groups. Some literature suggests a common belief from horse owners that horses stabled in box stalls are less prone to injury compared to those housed outdoors and in groups [39,40]. Qualitative research analyzing discussions between horse owners in internet forums discussed horse owner responses toward horse care as complex and temporal, observing a shift in horse owner priorities for their horse depending on the horse’s stage of life and use [41]. Horse owners may perceive the standard of care for their horse to be more complex than other welfare attributes related to physical health, mental well-being, and natural living, and this may contribute to the differences in agreement level between these three welfare attributes and the standard of care. These findings suggest that future research may aim to gather a more in-depth understanding of the discrepancies between horse owners’ knowledge of quality care and other horse welfare attributes. 

There was no significant association between owners’ attitudes toward horse welfare in indoor versus outdoor housing and whether their horses were kept outdoors full-time or indoors part-time. Therefore, it may be assumed that the belief that horse welfare is better when housed outdoors versus indoors was a factor that contributed to almost all horses in this study being kept outdoors for at least part of a 24 h period. However, the results also indicated that horse owners who disagreed with the statement that “the standard of care provided to horses is better when housed outdoors versus indoors” were more likely to house their horses indoors part-time. This may mean that some owners believed their horses’ standard of care to be better when housed indoors for at least part of the 24 h period. Researchers in Brazil reported that owners tended to keep more valuable horses in stalls and that some owners who board their horses may believe their horses’ standard of care to be better in stalls due to the higher cost compared to outdoor housing [8]. Similarly, Visser and van Wijk-Jansen [3] reported that Dutch owners understood that keeping horses in individual stalls compromises their welfare; however, despite this belief, between 52% and 84% of Dutch owners kept their horses in individual stalls full-time. A study in the UK reported that owners and trainers believed that housing horses in stalls for 24 h per day is acceptable due to cultural norms and a lack of scientific understanding [33]. Within Canada, Derisoud et al. (2016) [31] reported a discrepancy between horse owners’ and non-horse owners’ perception of Canadian horses’ allotted time outdoors, whereby non-horse owners believed that horses spend less time outdoors than reported by horse owners. Since most horses in this study were kept outdoors, with more exposure to public view, public perception potentially influenced horse owners’ decisions around horse care. Results of this study suggest there may be some discrepancies between horse owners’ ability to provide what they consider as good quality care and enhancing other aspects of their horse’s welfare. Understanding influencers surrounding horse owner decision-making will allow for more effective measures to support horse owners’ decisions regarding horse care.

Horse owners who disagreed more strongly with the welfare statements related to the horses’ “ability to live a natural life being better when housed individually versus in groups” and horses’ “physical health being better when housed individually versus in groups” were more likely to house their horses in groups. This result suggests that horse owners who keep their horses in groups may prioritize their horses’ natural living and physical health. Overall, some horse owners believe that they are sacrificing some degree of standard of care to enhance other aspects of their horse’s welfare, such as their mental well-being, physical health, and ability to live naturally. Owner attitudes have previously been shown to shape their decisions about their horse’s health [42,43]. Many factors influence owner attitudes toward horse welfare, such as their involvement in a lesson program [44]. Further, individuals who identify as female show often more concern for animal welfare [3,45] while a competitive motivation (i.e., utilitarian perspective) is associated with negative welfare attitudes [2,32]. The primary population of horse owners in this project identified as female and non-competitive. This may have contributed to the high number of individuals with attitudes and horse housing practices that enhance horses’ quality of life. Although not assessed in this study, and more research is recommended in this field, the specific equestrian discipline has been demonstrated to affect horse management practices [32,46]. Further, anthropomorphism or the attribution of emotions to animals can positively or negatively affect welfare in different contexts [47]; specifically, when behavior is misinterpreted or viewed from a lens that does not consider individual animals’ subjective experience [48,49]. Some degree of anthropomorphism may influence horse owners’ attitudes toward “good quality” care for horses. Understanding the horse owners’ perception of the relationship between their horse management strategies (i.e., the owners’ standard of care or environment) and their horse’s physical health, mental well-being, and their horse’s behavior will inform future strategies aimed at facilitating horse owners’ understanding of their horse’s well-being.

Owners discussed reasons for horse housing to improve aspects of their horse’s well-being. The sub-themes identified while inductively coding reasons and challenges owners face related to horse housing overlapped with animal welfare measures from the Five Domains Model [50]. The Five Domains Model was developed as a parallel to other animal welfare models (e.g., [14,51,52]) to capture and measure detailed nuances within specific domains of animal welfare (1. Nutrition, 2. Environment, 3. Health, 4. Behavior) as well as identify positive and negative mental states associated with each of the other four domains (5. Mental state). A recent study changed the fourth Domain from “Behavior” to “Behavior Interaction” to incorporate the effect of animals’ interaction with other animals, including humans, on their overall well-being [53]. This model has been used previously in the horse industry to understand equestrian experts’ perceptions of horse welfare-related issues [54] and assess horse owner perception of horse well-being [16]. Horse owners’ inherent understanding of the Five Domains Model suggests that this model may be helpful as a tool for information dissemination regarding horse care that is conducive to good welfare.

Owners in this study indicated housing their horses outdoors and in groups to prevent lameness and promote their horse’s health. It has been reported that housing horses in confined spaces such as individual stalls may lead to a higher risk of soft tissue injury when worked due to the inability of the horses’ muscles and tendons to maintain their elasticity with minimal daily movement [34,55,56,57]. Some owners in this study still regularly brought horses in for part of the day during the summer months. Further, keeping horses in groups was discussed as a challenge faced by owners. While group housing is generally beneficial for horses [6,58,59], important considerations for keeping horses in groups include ensuring the allocation of enough space and shelter for horses to avoid agonistic interactions [58] as well as the owners’ ability to appropriately match and introduce new horses within a group setting [38]. Owners also reported insect-related and manure management-related challenges, which could be potential reasons for part-time indoor housing during the summer months. Better manure management or other solutions to mitigate the disturbance from the bug population may allow horses to be outdoors more comfortably and reduce the amount of time required by owners to move horses throughout the day. Understanding the reasons behind owners’ decisions to keep horses indoors part-time will help facilitate systems to support owners caring for horses in a predominantly outdoor environment.

Overall, owners were generally satisfied with their horses’ living conditions. This finding suggests that keeping horses in outdoor, group living conditions has the potential to be satisfactory for both owners and their horses. However, horse owner satisfaction is not always indicative of their horses’ satisfaction. Due to each individual horse’s subjective and unique experience, an emphasis has been placed on validating animal-based welfare measures [60,61]. However, there appears to be a discrepancy between evidenced-based perception of animal behavior and owner/caretaker perception [62,63,64]. Luke et al. (2022) [36] argued that many scientists exploring horse behavior fail to acknowledge that a horse’s reality may differ from the owner’s experienced reality when caring for horses. An important future direction for the horse industry will be to lessen the gap between researchers’ and horse owners’ perceptions of good horse welfare.

Despite horse owners’ general satisfaction with their horse’s housing, horse owners still discussed some changes they would like to implement in their facilities. A study covering horse owner attitudes in Norway, Sweden, Denmark, and Finland determined that horse owners who kept their horses in groups were more satisfied with their horse’s housing than those who kept their horses individually [32], which is consistent with this study, whereby most horses were kept in groups followed by a majority of horse owners being satisfied or strongly satisfied with their horse’s housing. Dubois et al. (2019) [65] determined that Canadian horse owners were less likely to report structural problems with their facilities compared to management factors, such as stall cleanliness and manure management. In this study, it appeared that owners reported challenges related to their ability to provide care and challenges with their horse’s well-being but also reported that they wanted to implement structural and management-related changes in their facilities.

Some study limitations need to be considered. According to the latest estimate by Equine Canada (2010) [66], there were 4000 horses on 720 properties in PEI; hence, only approximately 8.3% of all farms and 5.3% of all horses on PEI were included in this study. Although horse owners across PEI participated, the self-selected basis of this study may indicate some bias in the horse owners who chose to participate (i.e., the population of horse owners in this study may not be generalizable to all PEI horses and horse owner populations). This paper focused on horse housing that included at least some form of outdoor access. Therefore, some bias may exist toward discussing outdoor access and group housing and less about the benefits and drawbacks of housing horses indoors full-time. Further, this lack of variation in the way horses were kept prevented the statistical comparison to attitudes of horse owners whose horses are housed indoors full-time. However, this study provides evidence that even among horse owners who provided some outdoor access within a 24 h period, there are statistical differences between owners’ attitudes who keep their horses outdoors full-time versus indoors part-time. Further, this paper only focused on horse housing during the summer and fall months, and thus, winter housing for some of the horses in the study is potentially different.

## 5. Conclusions

Horse owner attitudes toward keeping horses outdoors versus indoors and in groups versus individually appeared to reflect the way owners decide to keep their horses in PEI. However, compared to other welfare considerations, horse owners were less likely to agree that their horse’s standard of care is better when kept outdoors and in groups, suggesting some owners may believe there are trade-offs between their horse’s well-being and their ability to provide quality care for their horse. The findings highlight that reasons for housing horses outdoors and in groups were commonly related to enhancing their horse’s life rather than owner-centered preferences. This project suggests that housing horses in outdoor, group living conditions has the potential to be satisfactory for owners. Overall, understanding the perceptions of horse owners will provide insight regarding the best support and facilitation of practices that align with good horse welfare.

## Figures and Tables

**Table 1:** (a). Demographics of the horses (n = 212) in Prince Edward Island that were included in the study and owned by one of the owners in (b). Demographics of the horse owners (n = 76) in Prince Edward Island who participated in the study.

(a)		
	Horses (n = 212)	
Age (mean, median, range)	11, 11, <1–34	
Discipline (n, %)		
Non-competitive Western	44	21
Retired	43	20
Non-competitive English	24	11
School horse	21	10
Broodmare	17	8
Competitive Western	13	6
Competitive English	12	6
Standardbred racing	11	5
Young and developing	10	5
Other	17	8
Outdoor/Indoor Housing Type (n, %)		
Outdoor pasture/paddock	140	66
Indoor housing attached to outdoor pasture/paddock/run	31	15
Indoor	1	0.5
Moved between indoor housing and outdoor pasture/paddock	36	17
Moved between indoor housing attached to outdoor pasture/paddock/run and pasture/paddock	4	2
Individual/Group Housing Type (n, %)		
Grouped with horses	173	82
Grouped with other hoofed animals	2	1
Grouped with other horses and hoofed animals	24	11
Individually housed	13	6
(b)		
	Questionnaire Participants (n = 76)	
Age (mean, median, range)	47, 46, 21–81	
Gender (n, %)		
Woman	66	87
Man	10	13
Education (n, %)		
High school graduate	10	13
Some college	4	5
College diploma	25	33
Undergraduate degree	26	34
Postgraduate degree	11	15
Farm Type (n, %)		
Boarding facility	16	21
Boarding facility managed by participant	3	4
Home	40	52
Home and business	9	12
Other	8	11
Participants’ decision-making capacity		
I make all decisions by myself	30	40
I make decisions mostly by myself	17	22
I share the decision-making equally with someone else	27	36
Someone else is making the decisions	2	2
Number of Horses Owned (n, %)		
1 horse	24	32
2–4 horses	31	41
5–9 horses	13	17
10+ horses	8	11
Information Sources Utilized (n, %) ^1^		
Veterinarian	33	26
Other horse caretakers	27	22
Farrier	21	17
Published scientific literature	12	10
Other	9	7
Internet	7	6
Social media	7	7
Magazines	5	4
Newsletters	4	3

^1^ Question allowed multiple responses.

**Table 2:** Horse owners’ reasons for and challenges with housing horses in their current housing conditions. Responses are categorized by themes based on content analysis of open-ended questionnaire responses.

	Owner-Centered					Horse–Centered			
	Reasons		Challenges			Reasons		Challenges	
Subthemes	n	%	n	%	Subthemes	n	%	n	%
Ease of Care	27	71	N/A	N/A	Freedom to Perform Behavior	66	35	23	18
Reliance on Facility	6	16	N/A	N/A	Environment	35	19	77	61
Cost	3	8	N/A	N/A	Mental State	34	18	4	8
Tradition	2	5	N/A	N/A	Physical Health	32	17	8	6
Time Commitment	N/A	N/A	22	100	Nutrition	22	11	14	11

## Data Availability

The data presented in this study are openly available in FigShare at https://doi.org/10.6084/m9.figshare.21740534. Accessed on 11 January 2023.

## Supplementary Materials

The following supporting information can be downloaded at: https://www.mdpi.com/article/10.3390/ani13020275/s1, Supplement S1: Questionnaire.
