# Supplementary material for: Horse Housing on Prince Edward Island, Canada: Attitudes and Experiences Related to Keeping Horses Outdoors and in Groups
Source: Animals (Basel). 2023 Jan 13;13(2):275. doi: 10.3390/ani13020275 (PMC9855179; doi:10.3390/ani13020275)
Supplement: Supplementary file 1 [file animals-13-00275-s001.zip › animals-2135310-supplementary.pdf]

Supplement S1: Questionnaire

Ross et al. Horse Housing on Prince Edward Island, Canada: Attitudes and experiences related to keeping horses outdoors and in groups

Owner Demographics

Thank you so much for agreeing to take the survey.
First, we would like to collect some information about yourself.

What is your gender?

- Man (1)
- Woman (2)
- Gender not listed here (3)
- Prefer not to respond (4)

What year were you born?

____

What is your highest level of education?

- Less than high school degree (1)
- High school graduate (high school diploma or equivalent including GED) (2)
- Some college but no diploma (3)
- College diploma (2-year) (4)
- Bachelor's / Undergraduate degree (4-year) (5)
- Post-graduate or Professional degree (e.g. Master's, PhD, JD, MD, DVM) (6)

Animal Care Attitudes

Next, please indicate if you generally agree or disagree with the following statements.

Physical health

	Strongly disagree (1)	Disagree (2)	Slightly disagree (3)	Neither agree nor disagree (4)	Slightly agree (5)	Agree (6)	Strongly agree (7)
The **physical health** of horses is better when they are housed outdoors than when they are housed indoors (9)	o	o	o	o	o	o	o
The **physical health** of horses is better when they are housed individually than when they are housed in groups (10)	o	o	o	o	o	o	o

Mental wellbeing

	Strongly disagree (1)	Disagree (2)	Slightly disagree (3)	Neither agree nor disagree (4)	Slightly agree (5)	Agree (6)	Strongly agree (7)
The **mental wellbeing** of horses is better when they are housed outdoors than when they are housed indoors. (11)	o	o	o	o	o	o	o
The **mental wellbeing** of horses is better when they are housed individually than when they are housed in groups. (12)	o	o	o	o	o	o	o

Standard of care

	Strongly disagree (1)	Disagree (2)	Slightly disagree (3)	Neither agree nor disagree (4)	Slightly agree (5)	Agree (6)	Strongly agree (7)
The **standard of care** provided to horses is better when they are housed outdoors than when they are housed indoors (13)	o	o	o	o	o	o	o
The **standard of care** provided to horses is better when they are housed individually than when they are housed in groups (14)	o	o	o	o	o	o	o

Ability to live a natural life

	Strongly disagree (1)	Disagree (2)	Slightly disagree (3)	Neither agree nor disagree (4)	Slightly agree (5)	Agree (6)	Strongly agree (7)
The horses' **ability to live a natural life** is higher when they are housed outdoors than when they are housed indoors. (13)	o	o	o	o	o	o	o
The horses' **ability to live a natural life** is higher when they are housed individually than when they are housed in groups. (14)	o	o	o	o	o	o	o

Owner and Manager Care Practice Questions

Next, we are interested in how you care for the horses you own or manage.

Do you **own or manage** one or more horses? Select all that apply

☐ I own one or more horses (1)

☐ I manage one or more horses that I do not own (2)

☐ I do not manage or own horses (3)

Skip To: End of Survey If Do you own or manage one or more horses? Select all that apply = 3

Display This Question If:

Participant owns one or more horses

Please indicate the number of horses you **own:**

▼ 1 ... More than 10 horses

What best describes how you make decisions related to **how you house** the horses you own or manage?
For this survey, housing is defined as the primary place of residence for the horse.

○ I make all decisions by myself. (1)

○ I make decisions mostly by myself. (2)

○ I share the decision-making equally with someone else. (3)

○ Someone else is mostly making the decisions. (4)

○ Someone else is making all the decisions. (5)

Display This Question:

If participant owns 1 or more horses

What best describes how you make decisions related to **feeding** the horses you own or manage?

- ◯ I make all decisions by myself. (1)
- ◯ I make decisions mostly by myself. (2)
- ◯ I share the decision-making equally with someone else. (3)
- ◯ Someone else is mostly making the decisions. (4)
- ◯ Someone else is making all the decisions. (5)

Display This Question If:

Participants make at least some decision related to their horses housing or feed

What information sources do you use in your every-day housing and/or feeding related decisions for the horse(s) you own. Select all that apply:

- ▢ Other horse caretakers that I personally know (owners or managers) (1)
- ▢ Social Media (2)
- ▢ Newsletters (3)
- ▢ Magazines (4)
- ▢ Internet Forums (5)
- ▢ Veterinarian (6)
- ▢ Published scientific research (7)
- ▢ Farrier (8)
- ▢ Other (9)

Display This Question If:

If participant manages horses they do not own

For how many horses that you **do not** own are you making housing and/or feeding related decisions?

▼ 1 (1) ... 10+ (10)

Display This Question If:

If participant manages horses they do not own

In what capacity do you make housing-related decisions for the horse(s) you **do not** own? Select all that apply:

- ▢ I am the stable/farm manager (1)
- ▢ I am leasing a horse (2)
- ▢ I am a professional horse trainer (3)
- ▢ Other: (4) ____

Please indicate the names of up to 5 horses you OWN at this facility. If you both own and manage horses at this facility, please only list all horses you own first. If you do not own any horses at this facility, please indicate the names of up to 5 horses you MANAGE at this facility.

- ☐ Horse Name (1) ____
- ☐ Horse Name (2) ____
- ☐ Horse Name (3) ____
- ☐ Horse Name (4) ____
- ☐ Horse Name (5) ____

Horse Demographic Questions

Please indicate [Horse]'s age:

▼ Less than 1 (1) ... 42 (42)

Please indicate the primary types of exercise or discipline used for, [Horse]. Select all that apply:

- ☐ Retired or geriatric (1)
- ☐ Non-competitive (leisure) english type (2)
- ☐ Non-competitive (leisure) western type (3)
- ☐ English type School horse (4)
- ☐ Western type School horse (5)
- ☐ Competitive english type (6)
- ☐ Competitive western type (7)
- ☐ Racing (Standardbred, Thoroughbred, Quarter Horse) (8)
- ☐ Other (ex. Working horse, therapeutic, polo) (9) ____

Please describe the number of times [Horse] is fed hay per day, **in the last 4 weeks:**

- ◯ No hay fed, grass is sufficient source of roughage (1)
- ◯ Hay fed occasionally to supplement grazing on grass (2)
- ◯ 1 time per day (3)
- ◯ 2 times per day (4)
- ◯ 3 times per day (5)
- ◯ More than 3 times per day (6)
- ◯ Horse has free choice hay (ad libitum) (7)

X→

Does [Horse] receive concentrates to supplement his or her feeding regime **(in the last 4 weeks):**
In this survey, concentrate is defined as any type or mixture of grains, oats and/or minerals.

- ◯ Yes (1)
- ◯ No (2)

Display This Question If:

The participants horse receives grain

X→

Why does [Horse] receive concentrates? Select all that apply:

- ☐ Needs medication (1)
- ☐ For supplementation of vitamins and minerals (2)
- ☐ To increase calories to maintain physical health based on workload (3)
- ☐ To increase calories for weight gain (4)
- ☐ Doesn't need concentrates, but some is fed to reduce stress while other horses receive grain (5)
- ☐ To make them happy (6)
- ☐ Other: (7) ____

Horse Housing Type Questions

Please indicate how your horse(s) have been predominantly housed **during the last 4 WEEKS:**
The following questions will only focus on the horses housing within the last 4 weeks unless stated otherwise.

	**In Pasture/paddock** (3)	**In Indoor housing** *attached to* **pasture/paddock/run** (2)	**In Indoor housing** (1)	***Moved*** between indoor housing and pasture/paddock (4)	***Moved*** between indoor housing attached to outdoor run and pasture/paddock (5)
[Horse Name] (1)	○	○	○	○	○
[Horse Name] (2)	○	○	○	○	○
[Horse Name] (3)	○	○	○	○	○
[Horse Name] (4)	○	○	○	○	○
[Horse Name] (5)	○	○	○	○	○

Please indicate the type of farm [Horse(s)] is kept. Select all that apply:

- ☐ Training facility (1)
- ☐ Boarding facility that I manage (2)
- ☐ Boarding facility that is managed by someone else (3)
- ☐ At my home (4)
- ☐ Other: (5) ____

If all horses were housed identically, they would answer the questions in the following block for all horses. If not, all horses were housed identically, the "Housing Question" Block would be repeated for each horse.

Are the horses ([Horse Names]) housed identically to each other **during all seasons**?

- ○ Yes, they are housed exactly the same way (1)
- ○ No, they are not housed exactly the same way (2)

Housing Questions

Display This Question If Horse Housing Type is:

Indoor housing OR

Moved Between indoor housing and pasture/paddock

Please indicate how the horses ([Horse(s)]) are housed indoors **predominantly in the last 4 weeks,** select all that apply:

- ☐ Individual Stall (1)
- ☐ Indoor group housing shared with horses (2)
- ☐ Indoor group housing shared with other hoofed animals such as donkey(s), goat(s), cow(s), sheep (3)

Display This Question If Horse Housing Type is:

Indoor housing attached to pasture/paddock

Moved between indoor housing attached to outdoor run and pasture/paddock

Please indicate how the horses ([Horse(s)]) are housed **indoors (with attached pasture/paddock/run) pr**edominantly in the last 4 weeks, select all that apply:

- ☐ Individual stall with attached outdoor run (1)
- ☐ Indoor group housing with attached pasture/paddock shared with horses in the same pasture/paddock (2)
- ☐ Indoor group housing with attached pasture/paddock, shared with other hoofed animals such as, donkey(s), goat(s), cow(s), sheep (3)

Display This Question If Horse Housing Type is:

Pasture/paddock

Moved Between indoor housing and pasture/paddock

Moved between indoor housing attached to outdoor run and pasture/paddock

Please indicate how is the horses ([Horse(s)]) are housed specifically on the **pasture/paddock predominantly in the last 4 weeks**, select all that apply:

- ☐ Pasture/paddock not shared with other horses or hoofed animals (1)
- ☐ Pasture/paddock shared with horses in the same pasture/paddock (2)
- ☐ Pasture/paddock shared with other hoofed animals such as donkey(s), goat(s), cow(s), sheep (3)

Indoor versus Outdoor Housing

Display This Question If Horse Housing Type is:

Moved Between indoor housing and pasture/paddock

Approximately how many hours per day are the horses ([Horse(s)]) in each housing type:

- ☐ Hours in indoor housing (1) ________________
- ☐ Hours in pasture/paddock (2) ________________

Display This Question If Horse Housing Type is:

Moved between indoor housing attached to outdoor run and pasture/paddock

Approximately how many hours per day (24 hour period) do the horses ([Horse(s)]) spend in each housing type:

☐ Hours in indoor housing with outdoor access (1) ____

☐ Hours in pasture/paddock (2) ____

Display This Question If Horse Housing Type is:
Individual stall with attached outdoor run

Do the horses ([Horse(s)]) have access to the outdoor run all the time?

○ Yes (1)

○ No (2)

Display This Question If Horse Housing Type is:
Indoor group housing with attached pasture/paddock, shared with other horses and/or hoofed animals such as, donkey(s), goat(s), cow(s), sheep

Do the horses ([Horse(s)]) have access to the pasture/paddock all the time?

○ Yes (1)

○ No (2)

Display This Question if Horses in: Individual stall with attached outdoor run
do not have access to the attached outdoor run all of the time

Approximately how many hours per day (24 hour period) do the horses ([Horse(s)]) have access to the attached run:

☐ Hours with access to run **blocked** (1) ________________

☐ Hours with access to run **open** (2) ________________

Display This Question if Horses in: Indoor group housing with attached pasture/paddock, shared with other horses and/or hoofed animals such as, donkey(s), goat(s), cow(s), sheep

Do not have have access to the pasture/paddock all of the time

Approximately how many hours per day (24 hour period) do the horses ([Horse(s)]) have access to the attached pasture/paddock:

☐ Hours with access to pasture/paddock **blocked** (1) ________________

☐ Hours with access to pature/paddock **open** (2) ________________

Display This Question If Horse Housing Type is:

Pasture/paddock not shared with other horses or hoofed animals

While in pasture/paddock, how close are the horses ([Horse(s)]) in proximity to other horses?

○ Cannot see or touch other horses (1)

○ Can see other horses but cannot physically touch other horses (2)

○ Can physically touch other horses (any physical contact) (3)

Display This Question If Horse Housing Type is:

Individual stall

While in stall, how close are the horses ([Horse(s)]) in proximity to other horses?

- ◯ Cannot see or touch other horses (1)
- ◯ Can see other horses but cannot physically touch other horses (2)
- ◯ Can physically touch other horses (any physical contact) and has enough space to avoid contact from other horses (3)
- ◯ Can touch other horses and has minimal space to avoid contact from other horses (4)

Display This Question If Horse Housing Type is:

Individual stall with attached outdoor run

While in stall with attached outdoor run, how close are the horses ([Horse(s)]) in proximity to other horses?

- ◯ Cannot see or touch other horses (1)
- ◯ Can see other horses but cannot physically touch other horses (2)
- ◯ Can physically touch other horses (any physical contact) and has enough space to avoid contact from other horses (3)
- ◯ Can touch other horses and has minimal space to avoid contact from other horses (4)

Display This Question If Horse Housing Type is:

Individual stall

Please indicate whether the top portion of the door or window of the stall is able to open allowing the horses ([Horse(s)] to put their head out:

- Closed all the time (1)
- Closed most of the time (2)
- Open most of the time (3)
- Open all the time (4)

Display This Question If Horse Housing Type is:

Individual stall with attached outdoor run

Please indicate approximate dimensions of the run:

- Less than approximately 10ftx10ft (3mx3m) (1)
- Approximately 10ftx10ft (3mx3m) (2)
- more than approximately 10ftx10ft (3mx3m) (3)

Display This Question If Horse Housing Type is:

Indoor group housing with attached pasture/paddock shared with horses and/or other hoofed animals in the same pasture/paddock

Please indicate the approximate dimensions of the indoor group housing:

- Less than approximately 65ftx65ft (20mx20m) (1)
- Approximately 65ftx65ft (20mx20m) (2)
- Greater than approximately 65ftx65ft (20mx20m) (3)

Display This Question If Horse Housing Type is:

Indoor group housing with attached pasture/paddock

Please indicate the approximate dimensions of the pasture/paddock that is attached to the indoor group housing:

- ○ Less than approximately 65ftx65ft (20mx20m) (1)
- ○ Approximately 65ftx65ft (20mx20m) (5)
- ○ Between 65ftx65ft (20mx20m) and 1 acre (2)
- ○ Approximately 1 acre (4)
- ○ Greater than approximately 1 acre (3)

Display This Question If Horse Housing Type is:

Pasture/paddock

Moved Between indoor housing and pasture/paddock

Moved between indoor housing attached to outdoor run and pasture/paddock

Please indicate the approximate dimensions of the pasture/paddock:

- ○ Less than approximately 65ftx65ft (20mx20m) (1)
- ○ Approximately 65ftx65ft (20mx20m) (5)
- ○ Between 65ftx65ft (20mx20m) and 1 acre (2)
- ○ Approximately 1 acre (4)
- ○ Greater than approximately 1 acre (3)

Display This Question If Horse Housing Type is:

Indoor group housing shared with horses

Please specify the **total number** of horses that share the indoor group housing:

▼ 1 (1) ... 10+ (10)

Display This Question If Horse Housing Type is:

Indoor group housing shared with hoofed animals

Please specify the number of other hoofed animals the horses ([Horse(s)]) share the indoor housing with:

▼ 1 (1) ... 10+ (10)

Display This Question If Horse Housing Type is:

Indoor group housing shared with hoofed animals

Please indicate the type(s) of hoofed animal(s) the horses ([Horse(s)]) share the indoor group housing with:

____

Display This Question If Horse Housing Type is:

Pasture/paddock shared with horses

Please specify the **total number** of horses that share the pasture/paddock:

▼ 1 (1) ... 10+ (10)

Display This Question If Horse Housing Type is:

Pasture/paddock shared with hoofed animals

Please indicate the number of other hoofed animals the horses ([Horse(s)]) are housed with in the pasture/paddock:

▼ 1 (1) ... 10+ (10)

Display This Question If Horse Housing Type is:

Pasture/paddock shared with hoofed animals

Please indicate the type(s) of hoofed animal(s) the horses ([Horse(s)]) are housed with in the pasture/paddock:

____

Display This Question If Horse Housing Type is:

Indoor group housing with attached pasture/paddock shared with horses

Please specify the **total number** of horses that share the indoor housing with attached pasture/paddock:

▼ 1 (1) ... 10+ (10)

Display This Question If Horse Housing Type is:

Indoor group housing with attached pasture/paddock shared with other hoofed animals

Please specify the number of hoofed animals the horses ([Horse(s)]) share the indoor housing with attached pasture/paddock:

▼ 1 (1) ... 10+ (10)

Display This Question If Horse Housing Type is:

Indoor group housing with attached pasture/paddock shared with other hoofed animals

Please indicate the type(s) of hoofed animal(s) the horses ([Horse(s)]) are housed with in the indoor group housing with attached paddock:

____

Display This Question If Horse Housing Type is:
Pasture/paddock

Is there shelter provided in the pasture/paddock?

- ☐ No (1)
- ☐ Yes, man made type shelter (2)
- ☐ Yes, natural type shelter (3)

Display This Question If Horse Housing Type is:
Any form of group housing

Is there enough space for all animals to utilize the shelter at the same time?

- ○ Yes (1)
- ○ No (2)

*

Please specify **up to 3 reasons** why the horses ([Horse(s)]) housed the way they are, as opposed to an alternative type of housing

- ○ Reason 1 (1) ____
- ○ Reason 2 (2) ____
- ○ Reason 3 (3) ____

Please describe **up to 3 challenges** you face when housing the horses ([Horse(s)]) in their housing from the last 4 weeks:

- ◯ Challenge 1 (1) ____
- ◯ Challenge 2 (2) ____
- ◯ Challenge 3 (3) ____

Are the horses ([Horse(s)]) housed differently depending on the season?

- ◯ Yes (1)
- ◯ No (2)

Display This Choice if Horse Housing Type is not:
Indoor housing

- ◯ No, but horses are moved to indoor housing during extreme weather conditions (3)

Seasonal Housing Questions
All questions in this block repeat for each season depending on the participants response

Display This Question if:
Horses ARE Housed Differently Depending on the Season

Please indicate the season(s) where the horses ([Horse(s)] housing is different, select all that apply:

- ☐ Summer (4)
- ☐ Fall (1)
- ☐ Winter (2)
- ☐ Spring (3)

Display This Question if:

Horses are housed differently in the summer

During the summer, how are the horses ([Horse(s)]) housed?

- ○ Individual stall all the time (1)
- ○ Individual stall with run all the time (2)
- ○ Pasture/paddock all the time (3)
- ○ Indoor group housing all the time (4)
- ○ Indoor group housing with access to run all the time (5)
- ○ Splits time between stall and pasture/paddock (6)
- ○ Splits time between stall with attached run and pasture/paddock (7)
- ○ Splits time between indoor group housing and pasture/paddock (8)
- ○ Splits time between indoor group housing with attached run and pasture/paddock (9)

Display This Question if:

Horses are housed differently in the summer and splits time between stall and pasture/paddock

During the summer, please specify the number of hours the horses ([Horse(s)]) spend in the stall and pasture/paddock:

- Number of hours in stall (1) ________________
- Number of hours in pasture/paddock (2) ________________

Display This Question if:

Horses are housed differently in the summer and split time between indoor group housing and pasture/paddock

During the summer, please specify the number of hours the horses ([Horse(s)]) spend in the indoor group housing and pasture/paddock:

- Number of hours in indoor group housing (1) ________________
- Number of hours in pasture/paddock (2) ________________

Display This Question if:

Horses are housed differently in the summer and splits time between stall with attached run and pasture/paddock

During the summer, please specify the number of hours the horses ([Horse(s)]) spend in the stall with run and pasture/paddock:

- Number of hours in stall with attached run (1) ________________
- Number of hours in pasture/paddock (2) ________________

Display This Question if:

Horses are housed differently in the summer and splits time between indoor group housing with attached run and pasture/paddock

During the summer, please specify the number of hours the horses ([Horse(s)]) spend in the indoor group housing with run and pasture/paddock:

- Number of hours in indoor group housing with attached run (1) ________________
- Number of hours in pasture/paddock (2) ________________

Display This Question if:

Horses are housed differently in the summer and split time between stall with attached run and pasture/paddock or split time between indoor group housing with attached run and pasture/paddock

Do the horses ([Horse(s)]) have access to the run/pasture/paddock all the time?

- Yes (1)
- No (2)

Display This Question if :

Horses do not have access to the run all of the time

X→

Approximately how many hours per day (24 hour period) do the horses ([Horse(s)]) have access to the attached run:

- ☐ Hours with access to run **blocked** (1) ________________
- ☐ Hours with access to run **open** (2) ________________

Satisfaction Questions

You are almost done! The last section of this survey is to understand your general satisfaction/dissatisfaction with how your horse(s) are housed throughout the year.

How satisfied/dissatisfied are you with the housing of your horse(s)?

	Strongly dissatisfied (1)	Dissatisfied (2)	Neither satisfied nor dissatisfied (3)	Satisfied (4)	Strongly satisfied (5)
Your satisfaction with housing (1)	○	○	○	○	○

Display This Question if:

Horse owners choose anything under strongly satisfied

Please list **up to 3 housing changes you wish to make** in order of priority with 1 being the most prioritized and 3 being the least prioritized:

☐ 1. First priority for change (1) ____

☐ 2. Second priority for change (2) ____

☐ 3. Third priority for change (3) ____

Display This Question:

Please indicate the **barriers** that keep you from making the housing changes listed in the previous question:

☐ Barrier 1 (17) ____

☐ Barrier 218) ____

☐ Barrier 3 (19) ____

How satisfied/dissatisfied do you think the horse(s) are with how they are housed?

	Strongly dissatisfied (1)	Dissatisfied (2)	Neither satisfied nor dissatisfied (3)	Satisfied (4)	Strongly satisfied (5)
Horses satisfaction with housing (4)	o	o	o	o	o

Display This Question if:

Horse owners select anything less than strongly satisfied

Please list up to 3 things you think the **horse(s)** wishes to be different about the housing regime in the order of priority with 1 being the most prioritized and 3 being the least prioritized

- ☐ 1. First priority for change (1) ________________
- ☐ 2. Second priority for change (2) ________________
- ☐ 3. Third priority for change (3) ________________
